# Using a genetic test result in the care of family members: how does the duty of confidentiality apply?

**DOI:** 10.1038/s41431-018-0138-y

**Published:** 2018-04-27

**Authors:** Michael Parker, Anneke Lucassen

**Affiliations:** 1 Ethox Centre, University of Oxford, Oxford, UK; 2 Wellcome Centre for Ethics and Humanities, University of Oxford, Oxford, UK; 3 Clinical Ethics and Law, Faculty of medicine, University of Southampton, Southampton, UK; 4 Wessex Clinical Genetic Service, The Princess Anne Hospital, Southampton, MP105 LG PAH Coxford Road, Southampton, SO165YA UK

## Abstract

The use of genetic and genomic testing is becoming more widespread in healthcare and more inherited explanations for family history of diseases or conditions are being uncovered. Currently, relevant genetic information is not always used in the care of family members who might benefit from it, because of health professionals’ fears of inappropriately breaching another family member’s confidence. Such examples are likely to increase as testing possibilities expand. Here we present the case for use of familial information in the care and treatment of family members. We argue that whilst a clinical diagnosis in person A is confidential, the discovery of a familial factor that led to this diagnosis should be available for use in depersonalised form by health professionals to inform the testing and clinical care of other family members. The possibility of such use should be made clear in clinical practice at the time of initial testing, but should not require consent from the person in whom the familial factor was first identified. We call for further debate on these questions in the wake of high profile non-disclosure of genetic information cases, and forthcoming Data Protection legislation changes.

## Introduction

Consider the following case:

Helen (34) makes an appointment to see her doctor because she is worried about the possibility of developing breast cancer, and would like to know what screening or preventative measures she can take. She knows that there is a history of young onset breast cancer in her family but most of these relatives have died and she is not in contact with others who are still alive. In the past, she has been told that an accurate genetic test for her would require knowledge of the particular predisposing mutation in her family, but to Helen’s knowledge, no-one has been tested. Helen’s doctor also knows about the family history and she happens to look after several other family members. She knows the details of the disease-predisposing mutation in the family but also that the person in whom this was found (Helen’s aunt) said that she did not want her medical information to be shared with family members. The details of the mutation have therefore not been communicated to Helen, and Helen’s doctor is worried that if she were to use this information to guide testing in Helen, she would be breaching the aunt’s confidence.

But is there a duty of confidentiality to Helen’s aunt here? In this paper, we argue that whilst information about her, such as about the fact that she has had breast cancer, is confidential, the duty of confidence should not extend to the familial factors explaining this breast cancer. Close relatives have a greater degree of genetic code in common than unrelated people, and a genetic predisposition to disease may be common to several members of a family. Can a familial predisposition therefore be considered ‘personal and sensitive’ [1] when it is not identifying of any one person? We argue that such information should sometimes be available for use by health professionals in the clinical care of other family members whether or not there is consent from the person in whom it was first found.

### Background

Genetic information from family members can be important in the identification, treatment and counselling of patients at risk of inherited disorders. Its use is central to the risk assessment required to inform the selection of appropriate investigative tools or preventative options in the care of at-risk, unaffected family members. In Helen’s Aunt’s case, for example, her own access to testing will have depended to some degree upon the extent to which she was able to provide information about her family history. However, despite this, some patients do not subsequently share relevant information with other family members, and this can have an important impact on the possibility of effective care for these family members.

There are four main ways in which genetic information can fail to be communicated in families; (1) where a patient explicitly refuses to share genetic information with a family member for whom it might be useful; (2) where a patient, for one reason or another, simply never gets around to telling their relatives about it; (3) where a patient is overwhelmed by their own diagnosis and does not feel able to cope with sharing just yet; or, (4) where a patient is unaware of the fact that there are relatives who might benefit or has lost contact with relatives.

In many cases, the absence of information sharing may never come to light. However, in those cases where health professionals do have information relevant to others and know that this has not been communicated within a family, they often feel unsure about what they are permitted to do. In the case above, for example, Helen’s doctor has easy access to information that could provide important benefits and/or choices to Helen. Ought she to be able to use this information—in a depersonalised form—in Helen’s care if the patient in whom it was found has not herself told others about it, or has explicitly refused to allow this?

## Will genomics obviate the need to acquire or disseminate familial information?

One response to the issues presented by the familial relevance of genetic information might be to wonder whether this is less likely to arise in the era of genomics. Is it perhaps, a problem that is peculiar to rare inherited diseases before fast and cheap technologies examining an entire genetic code became available? This seems unlikely to be the case. There are good grounds for thinking that the wider use of genomics will increase rather than decrease the frequency with which such situations arise. For one, unless everyone’s genome is to be analysed (and interpreted) routinely early in life, most people at risk of a genetic condition will only come to know about it because of a diagnosis in themselves or in a relative. This means that decisions about testing and its prioritisation will continue to be strongly informed by familial information. Secondly, even if whole-genome analysis were to become routine, much variation would not be interpretable in the absence of signs, symptoms or a family history. Genomic findings do not, except in very rare cases, provide a definitive diagnosis or prognosis in the absence of phenotypic information, one key aspect of which will be family history information. This means that family history information will remain key to the clinical interpretation of genomic findings in many cases.

## The current situation

How are cases such as Helen’s currently dealt with? Notwithstanding the existence of guidelines encouraging a more familial approach to genetics [2], most practice continues to be framed by two strongly patient-centred principles: consent and confidentiality [3]. In this context, health professionals consider it permissible to use genetic information arising from a test on one of their patients for the care of relatives only if: (i) they have the consent of the patient to do so, or (ii) they come to a considered judgement that the failure to use this information would present a ‘risk of death or serious harm’ to a particular relative [4]. In reality, however, practice is much more restricted than this because even in situations where it would be widely agreed that this standard—a risk of death or serious harm—has been met, health professionals tend to be very risk-averse about breaching confidentiality. Helen’s GP might also have been confused by guidance that suggests that even in such cases ‘You should still seek the patient’s consent to disclosure’ [4].

This means that in practice, genetic information discovered in one person is currently usually treated as confidential, much as personal clinical information would be, and is only used in the care of others where there is explicit consent.

It is our view that in many cases, a duty of confidentiality should not prevent the use of familial genetic information in the care and treatment of family members. Although a familial factor may first be identified in a single individual, this in itself does not necessarily make it sensitive to, or identifying of, that person. Elsewhere, we have referred to the current framework in practice outlined above as the ‘personal account’ model of genetic information [5]. This approach has important advantages: High standards of confidentiality are seen as important because they enable patients to seek healthcare advice and treatment in the knowledge that this fact would not come to be more widely known. Confidentiality is a key contributor to the trust and confidence patients need in order to access care. A second advantage of the current approach is that it places a great deal of emphasis on respect for patient choices and values: their autonomy. Thirdly, maintaining high standards of confidentiality is important because patients have a reasonable expectation that this will be the case: the health system makes an implicit and sometimes explicit promise to treat patient information in certain ways. Finally, notwithstanding its emphasis on the importance of confidentiality in medicine, the current situation does allow information to be shared with family members without consent where there is a risk of death or serious harm.

The ‘personal account’ model does, however, have significant disadvantages. Perhaps the most important of these is the fact that significant numbers of people are currently missing out on genetic information that could improve their care. Those who miss out are at risk of being affected by an inherited condition but whose risk either does not meet the threshold of ‘risk of death or serious harm’ required in law for a legitimate breach of confidentiality, or clinicians find it difficult to judge whether this threshold has in fact been met.

A second weakness of the personal approach is that it does not adequately acknowledge the current dependence of good clinical practice on the collection and interpretation of familial information such as relatives’ health status to determine whether and which genetic testing should be done in a patient.

## An alternative approach

What might an alternative approach to the use of familial genetic information look like? Our preferred model would be one in which familial genetic information was available for the care of family members in situations where it has a significant potential for health benefit. That is, where a genetic test performed because of a family history of a condition has identified a familial factor and which is amenable to clinical use without necessarily identifying the person from whom it was obtained. We argue that a duty of confidentiality to one person is diminished if their result is potentially familial; the duty to use it in the care of others then outweighs a duty of confidentiality. Importantly, this is not to say that family members should be told about the health conditions or medical care of their relatives. The proposal is that in this setting, whilst clinical information about a person should, be kept confidential, information about the heritable predisposition that led to it should be available for health professionals to use in the care of family members.

What might be the advantages of such an approach? Clearly, the first and most important advantage is that family members would be in a position to take action against avoidable harms and obtain important health benefits that they would not have been able to access without this knowledge. It would, for example, mean that Helen would have access to a genetic test that would clarify whether or not she is at risk of inherited breast—and possibly other—cancers. This might, for example, enable her to be reassured that prophylactic surgery is of no benefit. An important additional advantage is that there is some evidence that this is what most people would be likely to want, i.e. a health service that was informed by all the relevant information [7, 6].

A third advantage is that it would address a current inequity in familial genetics; patients who are aware of a familial factor in their family are able to access care, but others who might also benefit from such knowledge are not. Furthermore, clinicians, such as Helen’s doctor, are left with information relevant to their patient that they feel unable to impart.

## Possible objections

There are a number of possible objections to our proposal. The first of these is that some might take the view that any familial genetic information derived from an individual is uniquely identifying—and hence confidential—even if depersonalised for familial use. Secondly, there may be some patients who would not want their test results to be used in the care of others,—even if it were not in itself identifying of them. It might be said that to do so in spite of their wishes would then be to fail to take their autonomy sufficiently seriously. A third criticism concerns the potential scale and scope of the duty of care such a model would create: would this imply the establishment of an open-ended duty of care to non-patients [7]? A fourth objection might be that public awareness of such uses of genetic information would impact on trust in the health professional patient relationship. A fifth potential criticism is that it might require significant resources and thus present a burden on already stretched health services.

## Can these objections be met?

Given the potential benefits of our proposed approach, it is important to consider whether these disadvantages could be addressed. We believe they can.

The first objection above is based on an empirical claim. The claim is that any genetic information derived from an individual is unique and identifying of that individual and hence confidential to them. This claim is simply false. In Helen’s case the harmful mutation is likely shared by several family members and not unique to the individual in which it was identified. Whilst it is true that the mutation may be unique to a particular family, it will not be unique to individual members of that family. The use of depersonalised information about a familial mutation for the care of others in the family will not necessarily be identifying of any individual within that family. In Helen’s case, for example, it is not necessary to tell Helen that a mutation was identified in her aunt. She could simply be told that the family history she already knows about is explained by a particular mutation that has been identified in her family. The objection above rests on the claim that depersonalisation is never or rarely possible. We do not believe this to be the case. Clearly there will be situations—perhaps paradigmatically in very small families in conflict—where the use of information might be identifying. Clinicians would need to pay attention to this possibility. The question of whether there is a risk of identification and how high this risk is, is of course an important judgement and one that requires careful professional consideration. We believe there will be many cases—like Helen’s—in which patients who are concerned about their familial risk could safely be offered a genetic test using information about a familial mutation without any confidentiality risk.

The second objection above, concerned respect for the autonomy of patients who do not want genetic information used in the care of family members even where they agree it would not be identifying. Our response to this is robust. We take the view that not wanting others to benefit from important health interventions without any reason is wrong.

The third criticism concerned the potential scale and scope of the duty of care such a model would create. We believe it would be possible, and justifiable, to address this concern through the adoption of a ‘response mode’ approach. That is, to adopt an approach in which familial genetic information would be available for use by professionals in situations where family members have approached health services because of a concern about an inherited condition. This would mean that Helen, for example, could be offered a predictive genetic test, but there would not necessarily be a duty to seek out all other family members who might potentially benefit (although Helen might in turn be encouraged to communicate with others).

We would welcome a debate about how such practices might be facilitated by national or international registers, but these more proactive approaches are beyond the scope of this paper in which we explore how the duty of confidentiality applies in such familial information. It is also important to note that there is an on-going debate about whether there ought to be a more active duty to warn in the context of familial information [8]. In this paper, we have not attempted to offer a legal analysis of confidentiality. However, it is interesting to note that the recent ABC case in the UK suggested initially that there was no legal duty to warn, although the court of appeal subsequently suggested that there may well be in certain clinical genetic situations [8, 9]. The approach we are proposing here is not one in which family members who have expressed no interest in knowing about their risk are to be identified and contacted; instead, it is one that operates in response mode, providing best available care for those family members such as Helen, who have presented with concerns about their inherited risk.

The fourth criticism of our approach is that it could lead to a reduction in trust in healthcare professionals: The reluctance of a patient to allow information derived from their genetic status to be used in the care of others might arise out of a genuine and well-founded worry about their confidentiality—even if the risk is very small—and that this might make patients less likely to come forward for treatment. Such worries are important to address and patients may need to be reassured about this. Partly the worry here is that patients might not have confidence in the depersonalisation process. They might be worried that it would be possible for family members to infer something about the clinical information of patients. This worry might lead to a reduction in trust even if the worry were to be unfounded. Whilst this is important to address, it is our view that a clear and convincing explanation is possible. Clinicians will need to explain, using examples, that clinical information, relating to an identifiable person and including, e.g. unique elements concerned with treatment decisions, can be kept distinct from depersonalised familial information that is not identifying so that this could be used without any breach of confidence at all. Interestingly, we are not alone in this belief. The UK’s General Medical Council, for example, acknowledges this possibility in its recommendations about genetic information which states ‘you should not disclose the patient’s identity in contacting and advising others about the risks they face’ [4]. This suggests that they too think it is possible to separate familial genetic and identifying ‘clinical’ information. This is important because we believe that if these protections are made explicit, many patients would want to see genetic information used in the care of family members, and would agree that where real benefits to family members are possible, these ought to be provided [10].

The fifth potential criticism of this approach is that it would require significant resources to store and make available such information and that these would have to be made at least on a national if not international level. Our view on this is that a judgement would need to be made about what would constitute reasonable record keeping practice in the context of constrained resources of publically funded healthcare systems. We consider that some relatively low resource steps could be taken immediately. For example, policies could be implemented whereby enquiries of other clinicians about results in relatives should be answered rather than blocked because of fears of confidentiality (as they currently might be). This might be combined with an expectation that genetic services working with families would have processes in place to ensure that such information was available for the care of the families to which they provide services if the above conditions are met.

## Conclusion

We have argued that a duty of confidentiality does not apply to the use of depersonalised familial genetic information in the care and treatment of other family members. There are strong arguments in favour of the adoption of the approach proposed here and convincing responses to the most important counterarguments. Familial information is not confidential to a person simply by virtue of the fact that it was identified in them. We have argued that where genetic information is clinically useful and where it does not raise substantive concerns about the identification of the individual from whom it was derived, it should be available for use in the care and treatment of family members. In cases where the current threshold for breaching confidentiality has been met, the proposed model would have the advantage of allowing health professionals to use such information e.g. the details of a familial breast cancer mutation without consulting or identifying the proband.
